# Electrical impedance tomography-guided prone positioning in a patient with acute cor pulmonale associated with severe acute respiratory distress syndrome

**DOI:** 10.1007/s00540-015-2084-y

**Published:** 2015-10-07

**Authors:** Toru Kotani, Hitoshi Tanabe, Hiroaki Yusa, Satoshi Saito, Kenji Yamazaki, Makoto Ozaki

**Affiliations:** Department of Anesthesiology, Tokyo Women’s Medical University, 8-1, Kawada-cho, Shinjuku-ku, Tokyo, 162-8666 Japan; Department of Cardiovascular Surgery, Tokyo Women’s Medical University, Tokyo, 162-8666 Japan

**Keywords:** Acute respiratory distress syndrome, Acute cor pulmonale, Prone positioning, Electrical impedance tomography

## Abstract

Electrical impedance tomography (EIT) is a noninvasive technique used to assess regional gas distribution in the lung. We experienced a patient with acute cor pulmonale during high positive-pressure ventilation for the treatment of severe acute respiratory distress syndrome. Prone positioning was beneficial for unloading the right ventricle for treatment of acute cor pulmonale. EIT played a role in detecting lung derecruitment at the patient’s bedside. Impedance distribution in ventral, mid-ventral, mid-dorsal, and dorsal layers before and 20 min after the start of prone positioning was 9, 48, 44, and 0 %, and 10, 25, 48, and 16 %, respectively. Lung recruitment monitored by EIT paralleled the improvement of PaO_2_/F_I_O_2_ from 123 to 239 mmHg. Timing of termination of prone positioning and ventilator settings such as lowering positive end-expiration pressure was determined to maintain dorsal recruitment as seen by EIT. The patient was weaned from mechanical ventilation on day 32 and discharged on day 200. EIT assessed the effects of prone positioning with real-time dynamic imaging and guided less injurious mechanical ventilation in a patient with acute cor pulmonale with dorsal lung derecruitment.

## Introduction

Acute respiratory distress syndrome (ARDS) is characterized by deleterious hypoxemia. Although high positive airway pressure is required to provide adequate gas exchange in patients with severe ARDS [1], it stresses pulmonary capillaries and increases pulmonary vascular resistance (PVR). It therefore creates right ventricular (RV) afterload and can cause RV failure. Acute cor pulmonale (ACP) is a severe form of RV failure caused by increased PVR or high pulmonary arterial pressure, signifying a poor prognosis for patients with ARDS [2–4].

Although prone positioning can be used to unload the RV in patients with ACP associated with ARDS [5], its application is not included in the standard care for ARDS patients because the indication is still controversial. Furthermore, it requires human resources and clinical experience to carry it out safely. Serious adverse effects, including pressure ulcers and accidental tube removal prevent caregivers from using it routinely. Additionally, the timing of initiation and termination of prone positioning is unclear.

Electrical impedance tomography (EIT) is a clinically available noninvasive technique that can be used to address the application and the timing of prone positioning. Its measurement principle is the creation of two-dimensional transverse single-slice images based on changes in impedance distribution originating from mechanical ventilation [6]. EIT can be used to assess the recruitment [7] and homogeneity [8] of gas distribution at the patient’s bedside.

We report a case in which EIT was beneficial for understanding lung derecruitment and assessing the effects of mechanical ventilation and prone positioning in a patient with ACP associated with severe ARDS.

## Case

A 77-year-old female was transferred from the general ward to the cardiac intensive care unit because of loss of consciousness, severe hypoxemia, and oliguria. She had undergone elective surgery for Y-graft replacement for an abdominal aortic aneurysm 1 month previously and emergency surgery for postoperative hemorrhage and necrosis of the sigmoid colon the next day. She had been treated with meropenem and vancomycin. She was immediately intubated and mechanically ventilated. On day 2, chest radiography demonstrated diffuse bilateral infiltration and her PaO_2_/F_I_O_2_ (P/F) was 123 mmHg without signs of cardiac failure, and she was diagnosed as having ARDS. Conventional mechanical ventilation (pressure controlled assist/control) with plateau pressure up to 28 cmH_2_O in combination with positive end-expiration pressure (PEEP) up to 15 cmH_2_O was applied in the first 20 h. However, SpO_2_ kept decreasing over time and the patient’s condition became more critical. The attending doctors decided to use airway pressure release ventilation (APRV) as a rescue mode. High PEEP levels were incrementally increased from 26 cmH_2_O. After APRV with F_I_O_2_ of 0.9 and high PEEP of 28 cmH_2_O was applied, gas exchange could be maintained for the next 2 days.

**Fig. 1:**
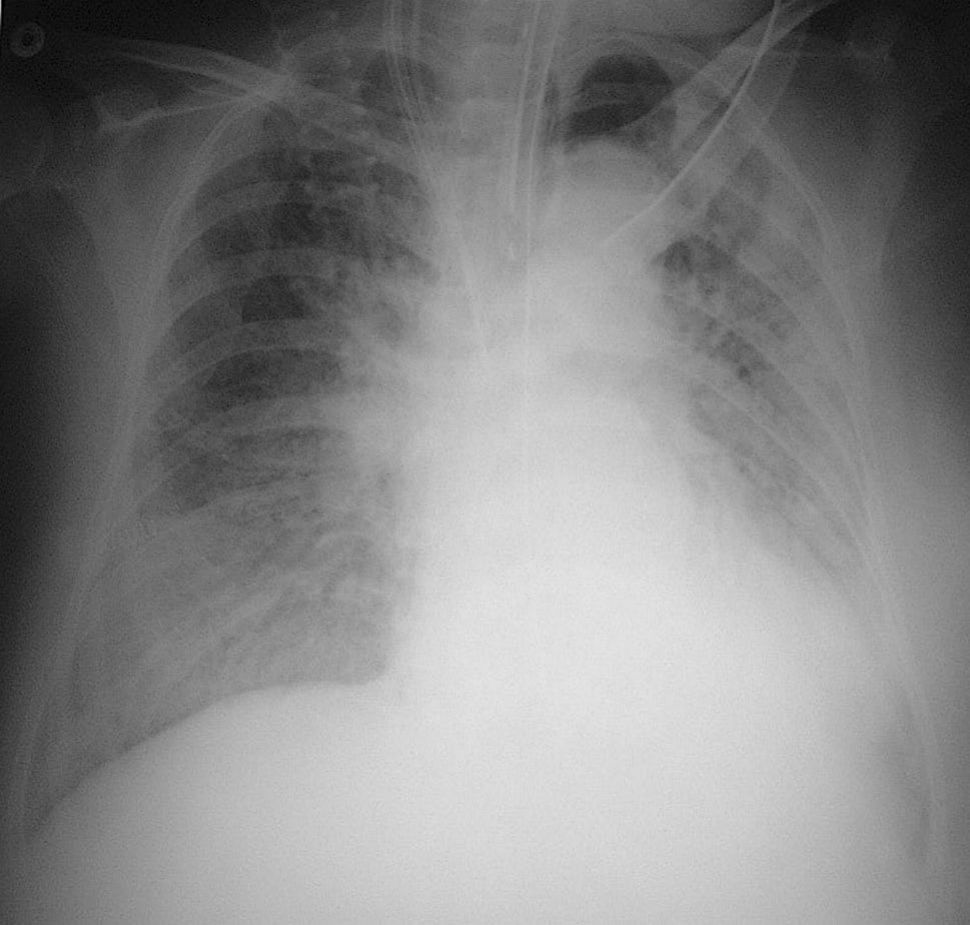
Chest radiograph on day 4, taken immediately after tracheal intubation

The cardiac surgeons consulted the intensive care doctors on day 4 regarding mechanical ventilation. The patient was in septic shock and was treated with a continuous infusion of epinephrine 0.15 μg/kg/min and norepinephrine 0.2 μg/kg/min (Fig. 1). As her hypoxemia and progressive hypercapnia had reached critical levels, we switched to inverse-ratio ventilation with an inspiratory-to-expiratory ratio of 2:1, inspiratory pressure of 34 cmH_2_O, PEEP of 18 cmH_2_O, and a ventilatory frequency of 31/min in order to increase minute ventilation without decreasing plateau pressure. Although the P/F ratio had stabilized (not worsened), the respiratory acidosis remained as indicated with PaCO_2_ at 62.9 mmHg and pH 7.209, respectively. Transthoracic echocardiography revealed severe RV dilatation (ratio between the RV and left ventricle [LV] end-diastolic areas was >1:1) in a four-chamber view, a paradoxical septal shift to the LV in the short-axis view, and severe tricuspid regurgitation, which were compatible with ACP [9]. The RV systolic pressure was estimated at >60 mmHg. The use of extracorporeal life support was excluded by the cardiac surgeons based on their expert opinion.

We proposed prone positioning for RV unloading. The doctors in charge hesitated because of lack of experience and reliable information concerning derecruitment of dependent lung regions.

**Fig. 2:**
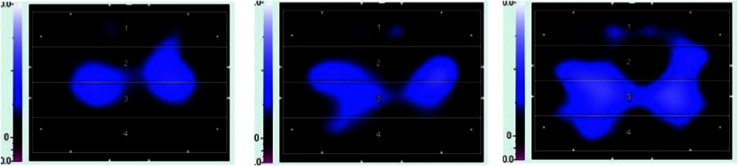
Images of electrical impedance tomography (EIT) at the end of inspiration before and after prone positioning. An image was divided into four layers from ventral to dorsal.The right lung is presented at the *left* side of the image. Regions with impedance changes of <10 % and >10 % of the determined maximum regional impedance change are represented in *black* and *blue*, respectively. As the impedance values increase, the *blue* turns *lighter blue*. A *white* color indicates the regions of maximum regional impedance change (i.e., 100 %) within the image. *Left*; before prone positioning trial, *center*; 20 min after the start of first trial, *right*; 20 min after the start of second trial

**Fig. 3:**
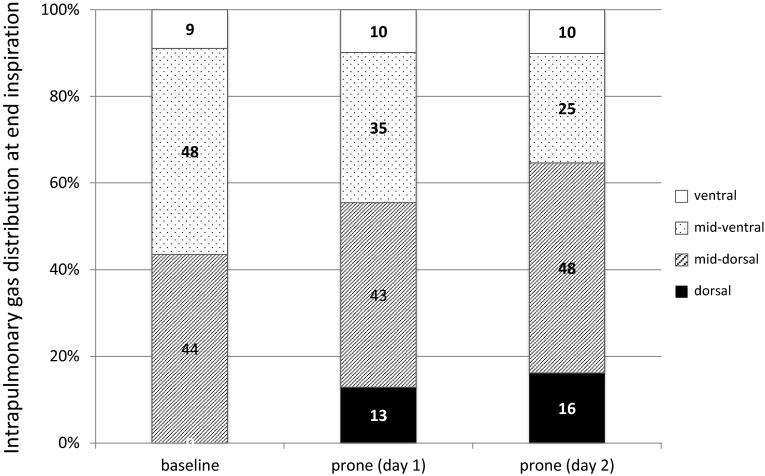
Intrapulmonary gas distribution at end-inspiration measured by electrical impedance tomography. The gas was shifted from the ventral to the dorsal area without changes in ventilatory settings

We then suggested using EIT (Pulmovista 500; Draeger Medical, Lübeck, Germany) to identify areas of lung derecruitment before prone positioning. Impedance values in each of the four layers (ventral, mid-ventral, mid-dorsal, dorsal) in the supine position were 9, 48, 44, and 0 %, respectively (Fig. 2, left). At 20 min after the start of prone positioning, 13 % of the total intrapulmonary gas had shifted from the mid-ventral to the dorsal layer with no changes in ventilator settings (Fig. 3); the change seemed to be occurring predominantly in the right lung (Fig. 2, center). The P/F, PaCO_2_, and pH improved an hour later to 239 mmHg, 36 mmHg, and 7.266, respectively. The first trial of prone positioning was discontinued at 3 h because of high doses of catecholamines and no change in the EIT images during the final 30 min. The RV systolic pressure according to transthoracic echocardiography was estimated at 47 mmHg. Blood cultures prepared on day 2 showed the presence of *Candida albicans* later on the same day as prone positioning was started, and micafungin 100 mg daily was started.

**Fig. 4:**
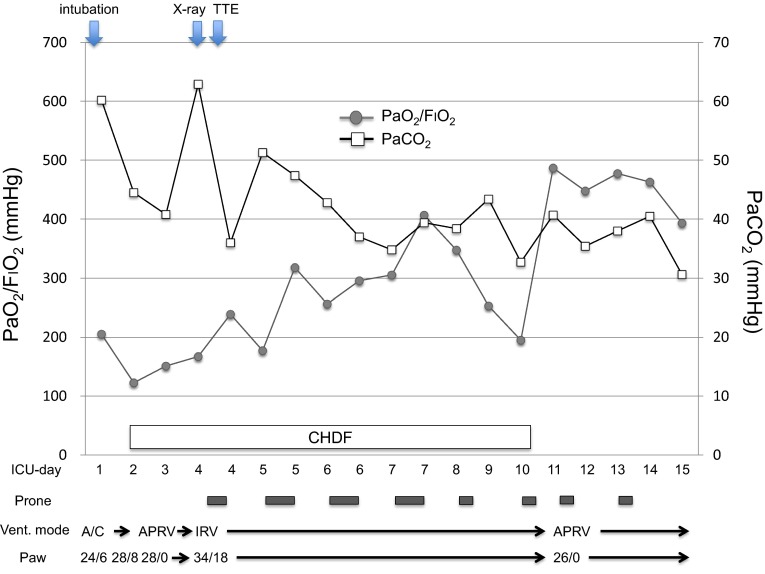
Time course of PaO_2_/F_I_O_2_ ratio, PaCO_2_, and ventilator settings at specific events and interventions. *A/C* assist/control mode, *APRV* airway pressure release ventilation, *IRV* inverse-ratio ventilation, *Paw* airway pressure (peak/PEEP in A/C and IRV, Phigh/Plow in APRV), *TTE* transthoracic echocardiography, *CHDF* continuous hemodiafiltration

The following day, the patient’s gas distribution had completely returned to baseline level. At 4 h after prone positioning was started, EIT imaging confirmed the presence of homogeneous gas distribution in almost all areas. Approximately 64 % of the gas was distributed in the mid-dorsal and dorsal layers (Fig. 2, right), in accordance with the increase in the P/F to 334 mmHg. The RV systolic pressure was estimated at 41 mmHg at 7 h after the end of prone positioning. Although the hemodynamics remained stabilized until day 9, airway pressures could not be reduced because of instability while the patient remained supine. Alveolar instability was observed as dorsal derecruitment on EIT with a PaO_2_ reduction from 434 to 195 mmHg. We switched to APRV with a *P* high of 26 cmH_2_O on day 10 to decrease the plateau pressure but maintain mean airway pressure. *P* high was determined at the lowest PEEP to maintain dorsal recruitment as seen by EIT. Prone positioning was continued for 14 days for periods of <7 h at each session (Fig. 4). No adverse effects associated with prone positioning or EIT imaging were observed. The patient was weaned from mechanical ventilation on day 32 and was discharged on day 200.

## Discussion

In our patient, candidemia caused septic shock, resulting in secondary ARDS. It has been reported that the incidence of ACP in ARDS varies from 22−50 % [2–4]. Additionally, ARDS patients with ACP showed a significantly higher mortality rate than ARDS patients without ACP (60 vs. 36 %, respectively) [2, 3]. We have been evaluating RV function in ARDS patients using echocardiography since 2007. The actual incidence of ACP is unknown, but intensivists should remember the impact of ACP and monitor RV function to protect against ACP. It has been reported that an elevated plateau pressure (≥27 cmH_2_O) [10], a driving pressure of ≥17 cmH_2_O [2], hypercapnia (PaCO_2_ ≥60 mmHg) [4], the severity of ARDS [2], and infectious causes [2] are related to the development of ACP. All of these factors, except the driving pressure, were found in our patient at the time of our initial examination.

The application of prone positioning played a role in this case. It augments lung recruitment and prevents overinflation [11]. ACP is prevented by decreasing the plateau and/or driving pressure and improving gas exchange, especially CO_2_ elimination [12]. Although the plateau and driving pressures could not be changed in our patient, the CO_2_ level dropped significantly after a 1-h trial of prone positioning. EIT showed the dorsal shift of intrapulmonary gas (Fig. 3) after prone positioning, and finally achieving homogeneous ventilation.

It is suggested that prone positioning reduces dead-space ventilation, resolves vasoconstriction, and decreases RV afterload. RV dilation was not observed in cases of successful high-frequency ventilation-recruited lungs but was observed if high-frequency ventilation failed [13]. During the first APRV trial, PEEP probably stressed the pulmonary circulation, but it could not be confirmed without serial echocardiographic RV monitoring. In the second APRV trial, EIT guided the lowest level of PEEP. It effectively kept the lungs open without deteriorating RV function. Longer prone positioning has been recommended [14], although each trial has been <7 h. This timing could be the reason for insufficient effects and the need for ventilator assistance. In our case, however, minimizing iatrogenic adverse events related to prone positioning was considered more important.

EIT played an important role in allowing prone positioning in the current case. Although computed tomography (CT) has been used to evaluate ventilation distribution [15], its clinical use is limited because of frequent radiation exposure, risk of transporting the patient, and lack of dynamic information. EIT, however, provides dynamic tidal images of gas distribution at the patient’s bedside. Furthermore, its radiation-free nature is advantageous. In addition, it enables frequent adjustment of the ventilator settings.

As recruitment parallels gas exchange improvement, as seen on EIT images, we were able to determine the correct ventilator settings and initiate and terminate prone positioning based on EIT data. Studies have reported that EIT provides useful information. A significant correlation between EIT and CT analyses of end-expiratory lung volume (EELV) during incremental and decremental PEEP trials was reported in pig saline lavage-induced acute lung injury models [7]. The authors also found EIT showed earlier derecruitment in dependent lung areas during the decremental trial, whereas the global tidal volume still continued to increase. Inspiratory-expiratory tidal volume changes after PEEP change estimated by EIT closely correlated with those measured by spirometry [16]. Good agreement was found between repeated EELV measurements and a simulated nitrogen washout technique to calculate lung volume [17]. EIT-guided mechanical ventilation preserved the alveolar architecture and maintained oxygenation and lung mechanics better than lower tidal volume ventilation in the saline lavage model [18].

In conclusion, EIT was used for detecting areas of derecruitment and assessing the effects of mechanical ventilation and prone positioning in a patient with severe ARDS and ACP.
